# Reactivity of Horse Sera to Antigens Derived From *Sarcocystis falcatula*–Like and *Sarcocystis neurona*

**DOI:** 10.3389/fvets.2020.573016

**Published:** 2020-11-02

**Authors:** Waléria Borges-Silva, Rogério F. de Jesus, Rachel Ferreira, Luís F. P. Gondim

**Affiliations:** Department of Anatomy, Pathology and Veterinary Clinics, School of Veterinary Medicine and Animal Science, Federal University of Bahia, Salvador, Brazil

**Keywords:** *Sarcocystis* sp., equine, antibody, Western blot, immunoblot

## Abstract

*Sarcocystis neurona* and *Sarcocystis falcatula* are protozoan parasites endemic to the Americas. The former is the major cause of equine protozoal myeloencephalitis, and the latter is associated with pulmonary sarcocystosis in birds. The opossum *Didelphis virginiana* is the definitive host of these parasites in North America. Four *Didelphis* species are found in Brazil, and in most reports in this country, *Sarcocystis* species shed by opossums have been classified as *S. falcatula*–like. It is unknown whether reports on *S. neurona*–seropositive horses in Brazil are also derived from exposure of horses to *S. falcatula*–like. The aim of this study was to test the sera reactivity of 409 horses in Brazil using antigens derived from a Brazilian strain of *S. falcatula–*like (Sarco-BA1) and from a North American strain of *S. neurona* (SN138). Samples were examined by immunofluorescent antibody tests (IFATs) at start dilutions of 1:20, and a selected number of samples was tested by Western blot (WB). Sera from 43/409 (10.5%) horses were reactive to *S. falcatula*–like and 70 of 409 (17.1%) were reactive to *S. neurona* antigen; sera from 25 animals (6.1%) were positive for both parasites by IFAT. A poor agreement was observed between the two employed IFATs (κ = 0.364), indicating that horses were exposed to more than one *Sarcocystis* species. Horse sera evaluated by WB consisted of four sera reactive to *S. falcatula*–like by IFAT, six sera positive to *S. neurona* by IFAT, two sera that tested negative to both parasites by IFAT, and a negative control horse serum from New Zealand. Proteins in the range of 16 and 30 kDa were recognized by part of IFAT-positive sera using both antigen preparations. We concluded that Brazilian horses are exposed to distinct *Sarcocystis* species that generate different serological responses in exposed animals. Antigens in the range of 16 and 30 kDa are probably homologous in the two parasites. Exposure of the tested horses to other *Sarcocystis* species, such as *Sarcocystis lindsayi, Sarcocystis speeri*, and *Sarcocystis fayeri*, or *Sarcocystis bertrami* cannot be excluded in the current study.

## Introduction

The coccidian parasite *Sarcocystis neurona* is the major cause of equine protozoal myeloencephalitis (EPM), a debilitating neurological disease that affects horses in the Americas (1, 2). The opossum *Didelphis virginiana* serves as definitive host of *S. neurona* in North America (3, 4), whereas *Didelphis albiventris* was identified as definitive host of *S. neurona* in Brazil (5). While only one species of *Didelphis* is found in North America, four species of this genus are found in Brazil: *D. albiventris, Didelphis aurita, Didelphis marsupialis*, and *Didelphis imperfecta* (6). The North American opossum (*D. virginiana*) is definitive host of three species of *Sarcocystis*: *Sarcocystis falcatula* (7), *S. neurona* (3), and *Sarcocystis speeri* (8). The South American opossum *D. albiventris* is definitive host of four species of *Sarcocystis*: *S. neurona, Sarcocystis lindsayi, S. speeri*, and *S. falcatula* (5, 9–11). Under experimental conditions, *S. falcatula* and *S. lindsayi* are infective for birds (11, 12), whereas *S. neurona* and *S. speeri* are infective for immunodeficient mice (13, 14).

Similarly to the protozoan parasite *Toxoplasma gondii, S. neurona* also contains several surface antigens (SAGs) which are probably associated with parasite virulence and host cell invasion (15). Three *S. neurona* SAGs (SnSAGs), named as SnSAG2, SnSAG3, and SnSAG4, were identified in merozoites of all *S. neurona* isolates and have been employed in an enzyme-linked immunosorbent assay (ELISA) for EPM in horses (16–18). Coding genes for SAGs from Brazilian isolates of *S. falcatula*–like are very similar to those from North American isolates of *S. neurona* (19). A high allelic variation is found for SAG2, SAG3, and SAG4 from *S. falcatula*–like, contrasting to SAGs from *S. neurona* in North America that possesses low genetic variation (20, 21).

Several serological techniques have been developed to detect *S. neurona* antibodies, including Western blot (WB), immunofluorescent antibody test (IFAT), *S. neurona* agglutination test and ELISA (2). WB using serum and cerebrospinal fluid from horses was the first serological test developed for EPM in horses (22). In the last two decades, IFAT and SnSAG ELISA have been validated for *S. neurona* infections and have been frequently used in veterinary practice and in research investigations (1). WB continues to be a valuable tool on *Sarcocystis* species investigations; however, its application has been essentially in research studies (2).

*S. falcatula* is a parasite shed by opossums that causes severe respiratory disease in birds (7, 23, 24). Serologic cross-reactivity between *S. falcatula* and *S. neurona* was suspected as some genes coding for immunodominant SAGs for these parasites are very similar (25). In this context, the SnSAG 2-4 ELISA would not be specific for *S. neurona* and could present cross-reactivity for *S. falcatula*–infected animals (25). In an experimental study conducted in the late 1990s, four horses in the United States did not seroconvert after experimental inoculation with *S. falcatula* sporocysts (26); it was assumed that cross-reactivity between *S. neurona* and *S. falcatula* would not be a concern when testing horses by SAG ELISA, as *S. falcatula* would not induce seroconversion in horses (1).

In Brazil, *Sarcocystis* species shed by opossums possess intriguing characteristics. In recent years, more than 50 *Sarcocystis* species isolates were obtained from Brazilian opossums, and almost 100% of them were infective to birds (budgerigars). However, these isolates were genetically distinct from both *S. falcatula* and *S. neurona* and, for this reason, classified as *S. falcatula*–like (20, 21, 27–29). The Brazilian isolates that were submitted for sequencing of SAG genes possessed a high allelic variation in their coding genes for SAG2, SAG3, and SAG4, which seems to represent genetic recombination between *S. neurona, S. falcatula*, or other unidentified *Sarcocsytis* species (19–21, 27–29). Based on this peculiar scenario in Brazil, we hypothesized that Brazilian horses may be exposed and seroconvert to other species of *Sarcocystis* shed by opossums, besides *S. neurona*. To address this hypothesis, we tested horse sera using antigen from a North American strain of *S. neurona* and antigen derived from a recently isolate of *S. falcatula*–like, which has been propagated in an avian cell line (27).

## Materials and Methods

### Horse Sera

Serum samples were obtained from 409 adult horses, including males and females, mostly from mixed breeds, and derived from Bahia and Rio Grande do Sul states in Brazil. Samples from Bahia state (n =217) were collected as part of the clinical screening for equine infectious anemia virus and for hematological checking. Samples from Rio Grande do Sul (n =192) were acquired in a commercial slaughterhouse for horse meat exportation. No animals were raised or handled for research purposes. Horse sera were stored for 5 years at −20°C in the Laboratory of Coccidian Protozoa at the School of Veterinary Medicine from Federal University of Bahia.

### Merozoites and Antigen Production

Antigens for IFATs and for WB consisted of merozoites of a North American strain of *S. neurona* (SN-138) (30) and merozoites from a South American strain of *S. falcatula*–like (27). *S. neurona* merozoites were propagated in Vero cells supplemented with RPMI-1650 + l-glutamine (Invitrogen/Gibco®, Carlsbad, USA), 1% antibiotic–antimycotic (100 units/mL of penicillin, 100 μg/mL of streptomycin, and 0.25 μg/mL of amphotericin B) (Gibco®, Carlsbad, USA), and 5% of inactivate bovine serum (Invitrogen/Gibco®, Auckland, NZ), at 37°C in a humidified incubator containing 5% CO_2_. *S. falcatula*–like merozoites were grown in the same conditions as described above, but instead of Vero cells, the parasites were cultured in a permanent chicken cell line (UMNSAH/DF-1) (31), as recently described (27). Cell monolayers containing merozoites of each parasite species were scrapped from the flasks, passed three times through a 26-gauge needle, filtered in Sephadex G-25 (GE Healthcare®) columns, and washed three times in phosphate-buffered saline (PBS) by centrifugation (1,500 g for 5 min).

### Immunofluorescent Antibody Tests

Volumes of 10 μL, containing of 3 × 103 purified merozoites of *S. neurona* or *S. falcatula*–like were added to each 5-mm well of IFAT slides, which were dried at 37°C. Antigen slides were immersed in cold acetone (−20°C) for 10 min for fixation and stored at −20°C until analysis. Antigen-coated slides were stored at a maximum time of 60 days until examination by IFAT.

Serum samples were tested at a starting dilution of 1:20 in PBS. Slides were incubated at 37°C for 30 min in a humid chamber and then washed for 10 min in a FA (fluorescent antibody) buffer (26.9 mM Na_2_CO_3_, 100 mM NaHCO_3_, 70.6 mM NaCl, pH 9.0) and 10 min in PBS and dried at 37°C. A fluorescein isothiocyanate–conjugated anti–horse immunoglobulin G (IgG) (Sigma–Aldrich®, St. Louis, USA) was used as secondary antibody at 1:32 dilution and incubated for 30 min in a dark and humid chamber. Slides were washed in FA and PBS as described above, dried at 37°C, and mounted with buffered glycerin. Reactions were observed under a fluorescent microscope (CiL, Nikon®). Positive controls consisted of sera from naturally exposed horses that reacted at 1:80 solely for each parasite (*S. neurona* or *S. falcatula*–like). Negative controls consisted of previously examined horse sera that tested negative for both parasites at dilutions <1:20. Positive reactions were characterized by full peripheral fluorescence of merozoites. Antibody titers were determined by double dilutions for all reactive sera.

### WB/Immunoblot

The term WB (Western blot) is used here and throughout the manuscript to indicate both WB and immunoblot. Cultured merozoites of *S. neurona* (4 × 10^7^ per membrane) or *S. falcatula*–like (2 × 10^7^ per membrane) purified in Sephadex G-25 columns (in *Merozoites and Antigen Production*) were pelleted by centrifugation and mixed with a reducing sample buffer (1% 2-mercaptoethanol, 2% sodium dodecyl sulfate (SDS), 7% glycerol, 48 mM Tris–HCl, pH 6.8), heated at 97°C for 10 min, and centrifuged at 13,000 g for 10 min at 4°C.

WB was performed similarly as previously reported (32). The solubilized proteins from merozoites were run on a 12.5% polyacrylamide gel electrophoresis with SDS. A prestained molecular weight marker with proteins from 10 to 180 kDa (PageRuler Prestained Protein Ladde, Thermo Scientific™) was used on each gel. Proteins were transferred from the gels to polyvinylidene difluoride (PVDF) membranes, blocked with PBS–Tween–gelatin (0.05% Tween 20 and 2% of gelatin) for 30 min, and stored at −20°C until analysis.

For immunoblot, each PVDF membrane coated with *S. neurona* or *S. falcatula*–like antigen was cut in 26 strips. Sera from 13 horses were selected for the analysis, including a negative control (horse serum from New Zealand), two horse sera that tested negative for both parasites by IFAT, six samples that tested positive for *S. neurona* by IFAT, and four sera that tested positive for *S. falcatula*–like by IFAT. Reactions were conducted in two different ways: 1) using antigen strips that were blocked with bovine serum containing antibodies to *Sarcocystis cruzi*, as reported by Rossano et al. (33); 2) using antigen strips not treated with anti–*S. cruzi* serum. Serum containing antibodies to *S. cruzi* was obtained by testing bovine sera with bradyzoites' antigen by IFAT (34).

Membrane strips from both parasites were blocked for 90 min with anti–*S. cruzi* bovine serum diluted at 1:65 (33) in PBS–Tween–gelatin. After five washings with 0.05% Tween-20 in PBS (PBS-T), membrane strips were incubated at room temperature with horse sera at 1:10 in PBS-T gelatin for 60 min. Then, they were incubated with anti–horse IgG peroxidase conjugate for 60 min and washed three times with PBS-T and three times with PBS. The reactions were revealed using diaminobenzidine peroxidase tablets and stopped by adding ultrapure water. The same 13 horse sera were tested by WB to both parasite antigens without initial incubation with anti–*S. cruzi* bovine serum.

### Statistical Analysis

To compare the percent agreement in IFATs for *S. neurona* and *S. falcatula*–like, Cohen κ statistics was used. Characterization of labeled bands in WB was determined by descriptive statistics, by means of the frequency of the observed bands.

## Results

### Immunofluorescent Antibody Tests

Antibodies to *S. neurona* (SN138 strain) and to *S. falcatula*–like (Sarco-BA1 strain) antigens were detected by IFAT in 17.1% (70/409) and in 10.5% (43/409) of the horses, respectively. A 1:20 dilution was used for the study. Simultaneous seropositivities for both antigens were observed in 6.1% (25/409) of the animals. A total of 322 (78.7%) of 409 horses were seronegative to both parasites. The agreement level for the two IFATs, expressed by the κ coefficient, was 0.364, indicating a fair agreement between the two tests. IFAT results are shown in Table 1. The maximum antibody titers observed for the two tested antigens was 1:160; 11 animals were positive at 1:160 for *S. neurona*, and 2 animals for *S. falcatula*–like presented titers of 1:160 by IFAT (Supplementary File).

**Table 1:** Seropositivity of Brazilian horses to *Sarcocystis neurona* and *Sarcocystis falcatula–*like tested by immunofluorescent antibody tests.

	***S. neurona***	***S. falcatula*–like**	***S. neurona* + *S. falcatula–like***
Frequency of seropositive	17.1% (70/409)	10.5% (43/409)	6.1% (25/409)
Frequency of seronegative	82.9% (339/409)	89.5% (366/409)	78.7% (322/409)

### Western Blot

In WB, reduced antigens from both parasites were tested using sera from 13 horses, including a negative control, two horses that tested negative to both parasites by IFAT, six horses that tested positive to *S. neurona*, and four horses that tested positive to *S. falcatula*–like by IFAT (Table 2).

**Table 2:** Horse sera selected for Western blot and their antibody titers for *Sarcocystis neurona* and *Sarcocystis falcatula* determined by IFAT.

**Animal ID**	**Antibody titer to *S. neurona***	**Antibody titer to *S. falcatula–*like**
1	Neg	Neg
2	Neg	Neg
3	Neg	Neg
4	1:160	Neg
5	1:80	Neg
6	1:80	Neg
7	1:80	Neg
8	1:80	Neg
9	1:80	Neg
10	Neg	1:40
11	Neg	1:20
12	Neg	1:40
13	Neg	1:20

**Figure 1:**
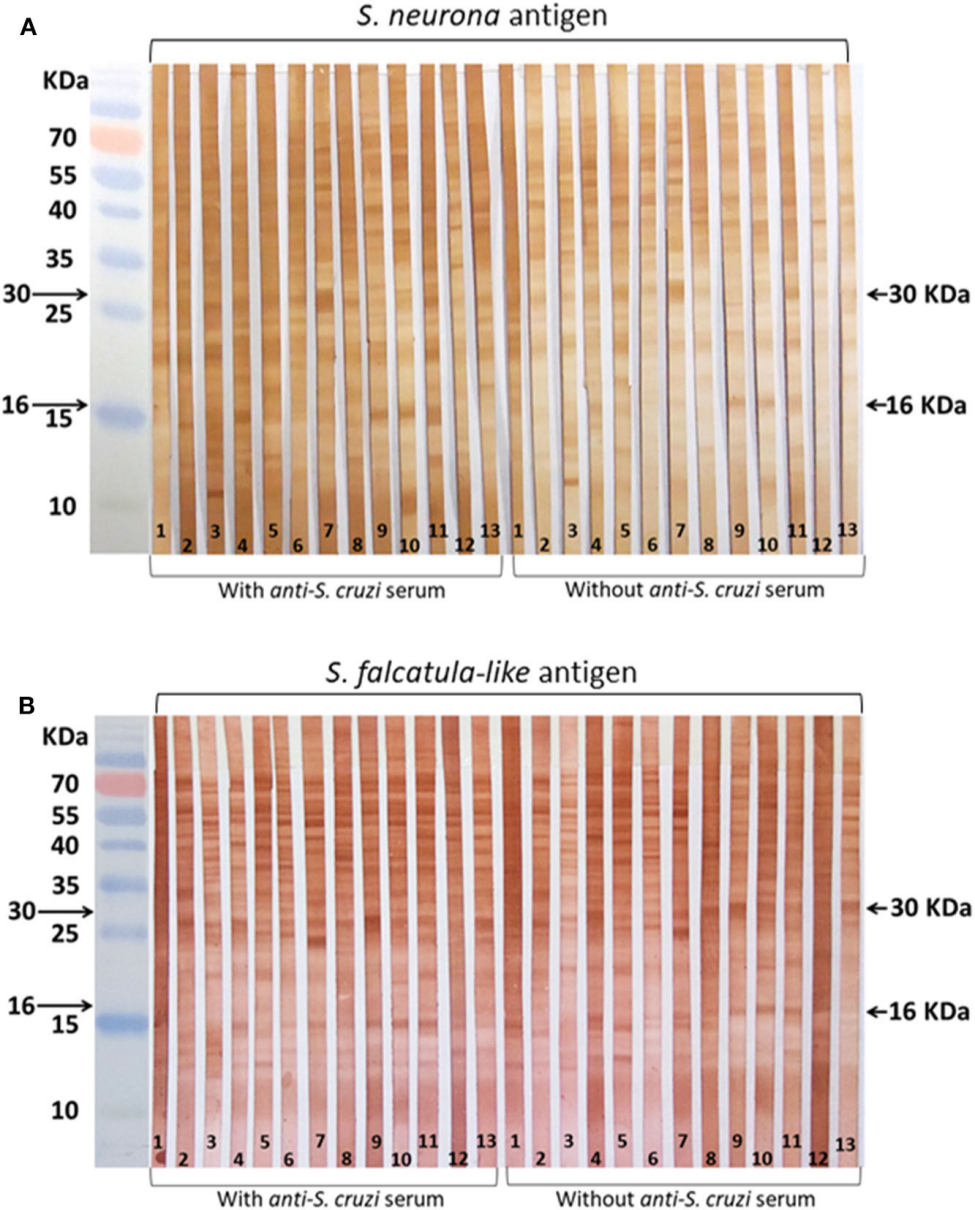
Horse sera tested by Western blot (WB). Serum samples were selected for WB by immunofluorescence antibody tests (IFATs). **(A)** Membrane strips coated with *Sarcocystis neurona* antigen; **(B)** membrane strips coated with *Sarcocystis falcatula–*like antigen. Reactions were conducted with and without blocking treatment with bovine anti–*Sarcocystis cruzi* serum. Strip 1: negative control (horse serum from New Zealand); 2–3: sera that tested negative for both parasites by IFAT; 4–9: sera that tested positive for *S. neurona* by IFAT; 10–13: sera that tested positive for *S. falcatula–*like by IFAT.

Most samples showed varying levels of reactivity to molecules in the regions of 16 and 30 kDa, regardless of *S. neurona* (Figure 1A) or *S. falcatula*–like (Figure 1B) were used as antigens in WB. The reactive bands in the antigen strips treated with anti–*S. cruzi* serum did not show significant differences from those not treated with anti–*S. cruzi* serum. Labeling of several antigens, besides those in the range of 16 and 30 kDa, were visualized between 10 and 70 kDa, with apparently no diagnostic value.

## Discussion

To test the hypothesis that Brazilian horses may be exposed and seroconvert to more than one *Sarcocystis* species shed by opossums, we examined horse sera from Bahia and Rio Grande do Sul states using two *Sarcocystis* species as antigens. Merozoites from a North American strain of *S. neurona* (SN-138) and from a South American strain of *S. falcatula–*like (Sarco-BA1) were employed as antigens in IFAT and WB. All samples were examined by IFAT, and 13 selected sera were tested by WB using antigens from both parasites. The sera tested by WB were processed with and without a blocking step with bovine serum anti–*S. cruzi*.

Results obtained by IFAT indicated that the examined horses reacted to more than one species of *Sarcocystis*; the κ coefficient was 0.364, supporting that the frequency of seropositivity in the IFAT for *S. neurona* had a fair agreement with the IFAT for *S. falcatula–*like. A total of 45 horses reacted solely to *S. neurona* antigen by IFAT, and 18 horses showed positive reactions solely to *S. falcatula–*like. A starting serum dilution of 1:20 was selected for the current study because in a previous work, a gold standard panel of horses infected with *S. neurona* had seropositivities between 1:20 and 1:80 by IFAT (35); these authors recommended a 1:80 cutoff in IFAT for horses suspected to have EPM. In the current work, equine sera were derived from horses with no neurological disease, and for this reason, we decided to use a less conservative cutoff.

The labeling patterns in WB using antigens of *S. neurona* and *S. falcatula–*like were very similar, indicating serological cross-reactivity for several shared antigens. The WB reactions using a blocking step with bovine anti–*S. cruzi* serum to minimize nonspecific reactions did not lead to any significant change in the results. Slight differences were observed in the intensity of the labeled antigens; however, it may be related to the time that enzymatic reactions were stopped during immunoblot. In literature, antigens regarded as immunodominant for *S. neurona* infection in horses possess approximate molecular weights of 16 and 30 kDa (33). Some authors also include proteins in the range of 7 to 10 kDa, besides the 16- and 30-kDa antigens as specific for *S. neurona* exposure (36). Positive reactions to one of the two proteins (16 or 30 kDa) are considered suspect for *S. neurona* infection (35). In subsequent studies with larger sample sets, the main concern of WB for *S. neurona* in horses corresponded to its low specificity (35, 37, 38).

Several reports on *S. neurona* infections or exposure in Brazilian animals have been conducted using North American strains of the parasite (39–43). It is worth to note that *S. neurona* antigen derived from Brazilian isolates of the parasite is not available for serological tests. The opossum species identified as definitive host for *S. neurona* in the United States (*D. virginiana*) does not exist in Brazil. The only description of *S. neurona* in a Brazilian opossum (*D. albiventris*) was reported in 2001 (5); parasite identification was mainly based on its infectivity to immunodeficient mice and by polymerase chain reaction–restriction fragment length polymorphism according to primers designed by Tanhauser et al. (44). At the time *S. neurona* was described in *D. albiventris* (5), the employed molecular tools were believed to precisely identify the *Sarcocystis* species infecting the opossum. In recent years, it became clear that additional molecular techniques are needed to differentiate *S. neurona* from closely related *Sarcocystis* species shed by opossums, including *S. falcatula–*like organisms (27).

In a recent publication, a Brazilian cat was reported to have *S. neurona* infection (45); based on the internal transcribed spacer 1 (ITS1) of the rDNA, this *Sarcocystis* species differed from organisms classified as *S. neurona* or *S. falcatula*. These authors used additional molecular markers, including *SAG* loci, *18S*, and *COX1*; the combined molecular data, mostly based on ITS1 and *SAG* loci, allowed the classification of this cat isolate as *S. neurona*, although it is clearly distinct from the North American isolates of *S. neurona* (45).

Studies on Brazilian *Sarcocystis* species shed by opossums, based on bioassay and different molecular markers, revealed that all isolates differed from *S. neurona*, in both biological and molecular aspects (19–21, 27, 29); the reported isolates were infective to budgerigars and possessed a high level of genetic recombination. In light of the peculiar scenario observed in Brazil, some questions have been raised by our research group: 1) May horses be infected with or present seroconversion to *S. falcatula–*like? 2) Are there other *Sarcocystis* species shed by opossums that are capable of infecting horses in Brazil? Although EPM associated to *S. neurona* was reported in Brazilian horses, identification of the parasite was mainly based on clinical, morphological, and immunological tests, including serology and immunohistochemistry (46, 47). So far, there is no isolation or molecular identification of *S. neurona* from any Brazilian horse. Serological studies in the country have been conducted with North American *S. neurona* antigens, in both IFAT and ELISA. In a serological survey performed with Brazilian horses from several states, the overall frequency of *S. neurona* antibodies was 69.6% (669/961) using an ELISA with SAG4 as antigen (40). Interestingly, in two recent seroepidemiological studies in Brazil using IFAT, frequencies of antibodies to the parasite were 26% (*n* = 506) in Minas Gerais (48), and 2.8% (*n* = 427) in the state of Alagoas (49); in these two studies, North American isolates of *S. neurona* were used with a 1:80 cutoff. In the present study, we detected 17.1% (*n* = 409) of seropositive animals by IFAT using *S. neurona* as antigen and 1:20 as cutoff. The differences in seropositivities between SAG4 ELISA (69.6%) and IFAT (2.8–26%) for Brazilian horses raise suspicions that results were overestimated by using SAG4 ELISA or underestimated by using IFAT. Inclusion of SnSAG ELISA (17, 18) in the current study, as well as a sample set of sera from horses with confirmed *S. neurona* infections, would highly aid on evaluation of cross reactions between anti–*S*. *neurona* antibodies and *S. falcatula–*like antigens. Performing immunoblotting with *Neospora* spp. antigens would also contribute to test potential cross-reactivity between this genus and *Sarcocystis* spp.

In the current work, we detected horses that reacted solely to *S. falcatula–*like antigen by IFAT and horses with positive reactions solely to *S. neurona* antigen by IFAT. For this reason, we suspected that more than one species of *Sarcocystis* species shed by Brazilian opossums induce seroconversion in horses. In a recent study, we performed experimental infection in Mongolian gerbils using *S. neurona* and *S. falcatula–*like (50). Serological cross-reactivity between the two parasites was clearly demonstrated by WB, whereas IFAT was able to distinguish infections caused by each of these parasites (50).

In conclusion, we demonstrated that using IFAT as serological test, Brazilian horses reacted differently to *S. neurona* and *S. falcatula–*like antigens. A 1:20 cutoff was employed in each IFAT; however, the optimal cutoff was not determined in this study, as no gold standard sera are available for Brazilian horses. Seroconversion of the tested animals to other *Sarcocystis* species that infect horses, including *Sarcocystis bertrami* or *Sarcocystis fayeri* (51), as well as to other *Sarcocystis* species shed by opossums, such as *S. lindsayi* or *S. speeri*, cannot be excluded in the current work. It is crucial to conduct further studies on molecular identification of *Sarcocystis* species in Brazilian horses with EPM, as well as to determine whether Brazilian strains of *S. falcatula–*like induce seroconversion in horses by oral ingestion of sporocysts of the parasite. To our knowledge, this is the first study to test horse sera with *S. falcatula–*like antigens and to provide evidence of serologic cross-reactivity in horses involving *S. neurona* and *S. falcatula–*like. Further studies are needed to determine an appropriate serological test to aid on diagnosis of EPM in Brazilian horses.

## Data Availability Statement

The original contributions presented in the study are included in the article/Supplementary Material. Further inquiries can be directed to the corresponding author/s.

## Ethics Statement

Ethical review and approval was not required for the animal study because no animals were raised or handled for research purposes, therefore, no license was required for the experiments. Samples from Bahia state (*n* = 267) derived from regular clinical screening for equine infectious anemia virus and for hematological checking. Samples from Rio Grande do Sul state (*n* = 142) were acquired in a commercial slaughterhouse for horse meat exportation.

## Author Contributions

WB-S: analysis and drafting of the manuscript. RJ: Western blot analysis and revision of the manuscript. RF: assistance in cell culture and IFAT. LG: designed the experiment and revised the manuscript drafts. All authors approved of the final version of the submitted manuscript.

## Conflict of Interest

The authors declare that the research was conducted in the absence of any commercial or financial relationships that could be construed as a potential conflict of interest.
