# Supplementary material for: Reactivity of Horse Sera to Antigens Derived From Sarcocystis falcatula–Like and Sarcocystis neurona
Source: Front Vet Sci. 2020 Nov 2;7:573016. doi: 10.3389/fvets.2020.573016 (PMC7667019; doi:10.3389/fvets.2020.573016)
Supplement: Supplementary file 1 [file Data_Sheet_1.docx]

Supplementary files

**Table 1.** Antibody titers to *Sarcocystis neurona* and *Sarcocystis falcatula*-like antigens in horses from Rio Grande do Sul state examined by immunofluorescent antibody test

| **Animal** | **Antibody titer to *S. neurona*** | **Antibody titer to *S. falcatula*-like** |
| --- | --- | --- |
| 1 | 1:80 | Neg |
| 3 | Neg | 1:40 |
| 4 | 1:160 | 1:40 |
| 9 | 1:160 | Neg |
| 11 | 1:160 | 1:80 |
| 13 | 1:40 | Neg |
| 16 | 1:160 | Neg |
| 17 | 1:160 | Neg |
| 18 | 1:80 | Neg |
| 23 | 1:40 | Neg |
| 26 | 1:40 | Neg |
| 28 | 1:20 | Neg |
| 29 | 1:40 | Neg |
| 36 | Neg | 1:20 |
| 61 | 1:40 | Neg |
| 76 | 1:40 | 1:40 |
| 77 | 1:40 | Neg |
| 79 | 1:160 | 1:40 |
| 80 | 1:20 | Neg |
| 85 | 1:80 | Neg |
| 86 | 1:80 | Neg |
| 90 | 1:40 | Neg |
| 93 | 1:40 | 1:40 |
| 95 | 1:80 | Neg |
| 98 | 1:80 | Neg |
| 99 | 1:160 | 1:40 |
| 103 | Neg | 1:20 |
| 106 | 1:40 | Neg |
| 109 | 1:20 | Neg |
| 110 | Neg | 1:20 |
| 111 | 1:40 | Neg |
| 113 | 1:80 | 1:40 |
| 116 | 1:80 | 1:40 |
| 117 | 1:160 | Neg |
| 118 | 1:80 | 1:80 |
| 121 | 1:80 | Neg |
| 125 | 1:80 | Neg |
| 126 | 1:40 | Neg |
| 128 | 1:80 | 1:160 |
| 129 | 1:80 | 1:80 |
| 138 | 1:160 | 1:160 |
| 147 | 1:80 | 1:40 |
| 155 | 1:80 | 1:80 |
| 156 | 1:40 | Neg |
| 158 | 1:20 | Neg |
| 162 | 1:160 | 1:40 |
| 165 | 1:40 | Neg |
| 166 | 1:20 | Neg |
| 167 | 1:160 | 1:40 |
| 169 | 1:80 | 1:40 |
| 172 | Neg | 1:40 |
| 176 | 1:80 | Neg |
| 178 | 1:80 | Neg |
| 181 | 1:20 | Neg |
| 182 | 1:20 | Neg |
| 184 | Neg | 1:40 |
| 186 | 1:20 | Neg |
| 192 | 1:80 | 1:80 |

Neg = Negative result

**Table 2.** Antibody titers to *Sarcocystis neurona* and *Sarcocystis falcatula*-like antigens in horses from Bahia state examined by immunofluorescent antibody test

| **Animal** | **Antibody titer to *S. neurona*** | **Antibody titer to *S. falcatula*-like** |
| --- | --- | --- |
| 7 | 1:20 | 1:20 |
| 8 | Neg | 1:20 |
| 19 | Neg | 1:20 |
| 34 | 1:20 | 1:40 |
| 35 | 1:20 | 1:20 |
| 40 | Neg | 1:20 |
| 48 | Neg | 1:20 |
| 63 | 1:20 | Neg |
| 66 | 1:40 | 1:40 |
| 74 | Neg | 1:20 |
| 85 | Neg | 1:20 |
| 104 | Neg | 1:20 |
| 111 | 1:20 | Neg |
| 112 | 1:20 | Neg |
| 114 | 1:20 | 1:20 |
| 118 | 1:20 | Neg |
| 121 | Neg | 1:20 |
| 123 | 1:40 | Neg |
| 124 | 1:40 | Neg |
| 125 | 1:20 | Neg |
| 128 | 1:20 | 1:20 |
| 137 | 1:40 | Neg |
| 142 | Neg | 1:20 |
| 145 | 1:20 | 1:20 |
| 146 | Neg | 1:20 |
| 148 | 1:20 | Neg |
| 154 | Neg | 1:20 |
| 177 | Neg | 1:20 |
| 183 | 1:20 | Neg |
| 208 | 1:20 | Neg |

Neg = Negative result
